# Epigenetic Dysregulation and Osteocyte Senescence: Convergent Drivers of Osteosarcopenia in Aging Bone and Muscle

**DOI:** 10.14336/AD.2025.0370

**Published:** 2026-05-25

**Authors:** Shahneela Nusrat, Rahman Ud Din, Muhammad Akram Tariq, Haisheng Yang

**Affiliations:** ^1^Department of Biomedical Engineering, College of Chemistry and Life Science, Beijing University of Technology, Beijing, China.; ^2^Higher Cancer Immunotherapy Center, Institute of Biomedicine and Biotechnology, Institute of Advance Technology (SIAT), Chinese Academy of Sciences, Shenzhen, China.

**Keywords:** Osteosarcopenia, Osteocyte Senescence, Epigenetic Remodeling, DNA Methylation, SASP, Senolytics, Bone-Muscle Crosstalk, Non-Coding RNAs, Oxidative Stress, Mitochondrial Dysfunction

## Abstract

Osteosarcopenia the concurrent deterioration of bone (osteoporosis) and muscle (sarcopenia) represents a critical yet understudied geriatric syndrome that synergistically amplifies frailty, fractures, and loss of independence in aging populations. This dual pathology imposes a staggering socioeconomic burden through increased disability, prolonged hospitalization, and elevated mortality. Despite its clinical urgency, therapeutic advances remain stagnant, as current interventions e.g., bisphosphonates, vitamin D supplementation are palliative and fail to address the shared molecular drivers of bone-muscle crosstalk. Emerging evidence implicates cellular senescence and epigenetic dysregulation as convergent mechanisms driving osteosarcopenia. Senescent osteocytes, burdened by oxidative stress and mitochondrial dysfunction, secrete pro-inflammatory cytokines e.g., IL-6, TNF-α and matrix-degrading enzymes (e.g., MMPs) via the senescence-associated secretory phenotype (SASP), which erodes bone integrity and propagates muscle atrophy. Simultaneously, epigenetic alterations DNA hypermethylation of osteogenic genes RUNX2, histone deacetylation repressing myogenesis MYOD1, and dysregulated non-coding RNAs (miR-133, miR-214) lock musculoskeletal tissues into a degenerative state. These processes are exacerbated by age-related inflammaging and metabolic disturbances e.g., NAD+ depletion, which amplify oxidative stress and chromatin instability. The synergy between senescence and epigenetics in perpetuating osteosarcopenia remains poorly defined. Most preclinical models overlook comorbidities (e.g., diabetes, chronic inflammation) that accelerate musculoskeletal decline. Current therapies senolytics, histone deacetylase (HDAC) inhibitors lack tissue specificity and exhibit pleiotropic effects. This review addresses these gaps by synthesizing cutting-edge insights into the senescence-epigenetics axis as a unifying driver of osteosarcopenia. By elucidating how SASP factors (e.g., myostatin) and epigenetic reprogramming e.g., sirtuin 1 (SIRT1) hypermethylation disrupt bone-muscle crosstalk, we propose novel strategies to break the self-sustaining cycle of tissue degeneration. We highlight the promise of precision geroscience leveraging CRISPR-engineered organoids, multi-omics profiling, and AI-driven biomarkers to decode tissue-specific vulnerabilities and design dual-target therapies e.g., senolytics ++ bromodomain and extra-terminal (BET) inhibitors.

## 1. Introduction

Age-related bone loss (osteoporosis) and muscle deterioration (sarcopenia) increasingly coexist as osteo-sarcopenia, amplifying frailty, fracture risk, and disability in older adults [1, 2]. By 2050, the global population over 60 is projected to reach 2.1 billion, with osteoporosis affecting over 200 million individuals and sarcopenia impacting up to 50% of those over 80 [3]. These conditions cause muscle weakness and elevate fall risk, while bone fragility heightens fracture-related complications like immobility [4]. The pathophysiology of osteosarcopenia involves shared mechanisms, including chronic inflammation, hormonal changes e.g., declines in estrogen, testosterone, and vitamin D, and mitochondrial dysfunction [5]. For instance, elevated pro-inflammatory cytokines like IL-6 and TNF-α accelerate muscle protein breakdown and inhibit bone formation, while age-related declines in growth hormone and IGF-1 impair tissue repair [6]. Notably, senescent osteocytes and muscle cells accumulate with aging [7], secreting harmful factors that disrupt tissue repair and homeostasis [8].

Osteocytes, central regulators of bone-muscle crosstalk, exhibit age-related dysfunction through oxidative stress and increased sclerostin production, which suppresses bone formation [9]. They also communicate via extracellular vesicles carrying microRNAs e.g., miR-483-5p that impair muscle metabolism [10]. Epigenetic dysregulation further links skeletal-muscle aging. Hypermethylation of osteocyte reduces collagen synthesis genes like *COL1A1*, while elevated histone deacetylase (HDAC) activity in muscle represses mitochondrial biogenesis e.g., *PGC-1α* [11, 12]. Epigenetic clocks, which track DNA methylation changes, correlate strongly with musculoskeletal decline, offering biomarkers for early intervention [13]. Promising therapies include senolytics (e.g., dasatinib) to clear senescent cells, HDAC inhibitors e.g., vorinostat, and exercise, which remodels DNA methylation e.g., *MYOD1* activation in muscle stem cells [14, 15].

## 2. Osteocyte Senescence in Bone Aging

### 2.1 Mechanisms Driving Osteocyte Senescence

Osteocyte senescence, a hallmark of skeletal aging, is driven by cumulative oxidative stress and DNA damage [16]. Reactive oxygen species (ROS), generated endogenously through mitochondrial dysfunction or exogenous factors such as radiation and toxins, overwhelm antioxidant defenses including superoxide dismutase and glutathione in aging osteocytes, leading to macromolecular damage [17]. Oxidative stress directly damages nuclear and mitochondrial DNA, triggering double-strand breaks (DSBs) that activate the DNA damage response (DDR) pathway, including ATM/ATR kinases and p53/p21 signaling [18]. Persistent DDR activation forces osteocytes into senescence, characterized by irreversible cell-cycle arrest and metabolic dysregulation [19]. For example, aged osteocytes exhibit elevated γH2AX foci a marker of DSBs and reduced repair capacity, correlating with bone loss in murine models [20] (Table 1). Furthermore, mitochondrial ROS production in osteocytes disrupts autophagy, exacerbating the accumulation of damaged organelles and proteins [21]. Interventions such as N-acetylcysteine (NAC), a ROS scavenger, or mitochondrial-targeted antioxidants e.g., MitoQ have shown promise in reducing oxidative stress and preserving osteocyte viability in preclinical studies [22]. However, age-related declines in endogenous antioxidants like SOD2 and catalase limit cellular resilience, highlighting the need for therapeutic strategies to enhance redox homeostasis in aging bone.

**Table 1:** Mechanisms of Osteocyte Senescence and Their Impact on Bone-Muscle Crosstalk.

Mechanism	Key features	Bone	Muscle
Oxidative Stress	ROS accumulation, mitochondrial dysfunction, impaired autophagy	DNA damage, reduced osteocyte viability, sclerostin upregulation	Muscle satellite cell dysfunction, atrophy
DNA Damage Response	γH2AX foci, ATM/ATR-p53/p21 activation, cell-cycle arrest	Persistent senescence, trabecular bone loss	Impaired muscle repair, reduced regenerative capacity
SASP Secretion	IL-6, TNF-α, MMPs, RANKL, EVs with miR-34a	Osteoclast activation, collagen degradation, suppressed osteoblast activity	Myostatin/TGF-β-driven satellite cell inhibition, muscle wasting
Mechanotransduction Loss	Reduced connexin 43, sclerostin-mediated Wnt inhibition	Impaired load sensing, reduced bone formation	Disrupted mechanical signaling, muscle weakness

**Figure 1.**
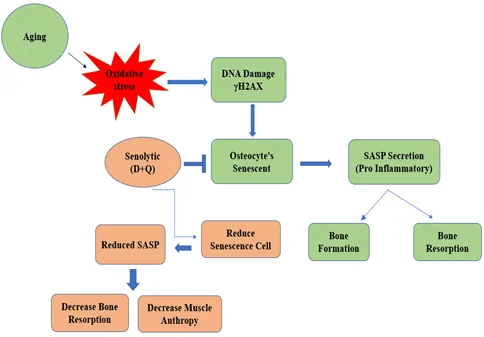
**Mechanisms of Osteocyte Senescence and Senescence-Associated Secretory Phenotype (SASP)**. This figure represents oxidative stress and DNA damage drive senescence, triggering pro-inflammatory SASP. Senolytics (dasatinib/quercetin) clear senescent cells, reducing bone resorption and muscle atrophy.

The Senescence-Associated Secretory Phenotype (SASP) disrupts bone remodeling by amplifying senescence through pro-inflammatory mediators secreted by senescent osteocytes, including IL-6, IL-1β, TNF-α, matrix metalloproteinases (MMPs), and RANKL. For example, IL-6 synergizes with RANKL to accelerate osteoclastogenesis (osteoclast formation) and suppress osteoblast function, while MMPs independently degrade collagen. These combined actions enhanced osteoclast activity, impaired osteoblast activity, and collagen breakdown collectively destabilize bone homeostasis. This mechanism underscores how SASP components like IL-6, RANKL, and MMPs drive pathological bone remodeling through distinct yet coordinated pathways [23, 24]. SASP also induces paracrine senescence, spreading damage to neighboring cells via extracellular vesicles (EVs) carrying microRNAs e.g., miR-34a and oxidized lipids [25]. Additionally, SASP factors like myostatin and TGF-β contribute to sarcopenia by inhibiting muscle satellite cell differentiation, linking bone aging to muscle atrophy [26]. Therapeutic approaches targeting SASP include senolytics e.g., dasatinib and quercetin, which eliminate senescent osteocytes and improve bone density in aged mice [27, 28], and SASP-neutralizing strategies, such as IL-6 inhibition or MMP-13 blockade, which show potential for treating osteosarcopenia [29] (Fig. 1). However, SASP’s dual role in tissue repair and inflammation necessitates balanced therapeutic strategies to mitigate its detrimental effects without compromising beneficial functions.

### 2.2 Consequences of Senescent Osteocytes on Bone Homeostasis

Senescent osteocytes profoundly disrupt bone remodeling by impairing their dual role as mechano-sensors and regulators of bone turnover [30]. Mechanotransduction, the process by which cells convert mechanical forces (e.g., fluid shear stress) into biochemical signals, is impaired in senescent osteocytes due to sclerostin-mediated Wnt inhibition and connexin-43 dysfunction [31]. In healthy bone, osteocytes detect mechanical strain through fluid shear stress within the lacunar-canalicular network, triggering signaling pathways e.g., Wnt/β-catenin that promote osteoblast-mediated bone formation and inhibit osteoclast activity [32]. However, senescent osteocytes exhibit elevated secretion of sclerostin, a Wnt inhibitor, and RANKL, an osteoclast-activating factor, which skews the balance toward excessive resorption and reduced formation [33]. For example, aged mice with accumulated senescent osteocytes show a 40% increase in sclerostin expression, correlating with trabecular bone loss [33, 34]. Additionally, senescence diminishes connexin 43 gap junction function, critical for transmitting mechanical signals to osteoblasts, thereby impairing adaptive responses to load [35, 36]. This loss of mechanosensation exacerbates skeletal fragility, as bones fail to reinforce structurally vulnerable areas. Preclinical studies demonstrate that clearing senescent cells with senolytics (e.g., dasatinib + quercetin) restores mechanotransduction and improves bone strength in aged mice, highlighting the therapeutic potential of targeting senescent osteocytes [37].

Senescent osteocytes orchestrate a toxic microenvironment by secreting SASP factors that dysregulate osteoclast and osteoblast activity [38]. Pro-inflammatory cytokines such as IL-6 and TNF-α amplify osteoclastogenesis by upregulating RANKL and downregulating osteoprotegerin (OPG), a decoy receptor for RANKL [39]. Concurrently, SASP-derived MMPs degrade collagen, releasing embedded growth factors (e.g., TGF-β) that further stimulate osteoclast recruitment [40]. Conversely, osteoblast function is suppressed through Wnt pathway inhibition (via sclerostin and DKK1) and oxidative stress-induced apoptosis [41]. Senescent osteocytes also secrete extracellular vehicles (EVs) containing pro-osteoclastic microRNAs e.g., miR-214, which enhance osteoclast precursor differentiation [42]. In co-culture experiments, senescent osteocytes increase osteoclast resorption pits by 70% while reducing osteoblast mineralization by 50% [30]. Therapeutic interventions, such as anti-IL-6 antibodies or sclerostin-neutralizing agents (e.g., romosozumab), have shown efficacy in restoring bone homeostasis in preclinical models [43]. However, the pleiotropic nature of SASP complicates clinical translation, necessitating strategies to selectively block detrimental signals while preserving beneficial ones.

## 3. Epigenetic Remodeling in Age-Related Skeletal and Muscular Decline

### 3.1 DNA Methylation Dynamics in Bone Integrity

DNA methylation, a critical epigenetic mechanism involving the addition of methyl groups to cytosine residues in CpG regions, regulates gene expression patterns essential for bone development, homeostasis, and aging [44]. Age-related epigenetic drift the progressive accumulation of stochastic DNA methylation changes disrupts transcriptional programs critical for bone homeostasis [45]. This dynamic process is governed by the opposing actions of DNA methyltransferases (DNMTs), which catalyze methylation, and ten-eleven translocation (TET) enzymes, which facilitate demethylation [46]. During aging, dysregulation of DNMT and TET activity disrupts the epigenetic landscape of bone cells, contributing to skeletal fragility. DNMTs, including the maintenance enzyme DNMT1 and the de novo methyltransferases DNMT3A/B, are vital for preserving methylation patterns that govern osteoblast differentiation and bone formation [46]. Aging is associated with locus-specific hypermethylation at promoters of osteogenic genes, such as RUNX2, a master regulator of osteogenesis [47] (Figure 1). Hypermethylation of RUNX2 in aged mesenchymal stem cells (MSCs) suppresses its expression, impairing osteogenic potential [48]. Similarly, age-related overexpression of DNMT3B in osteoblasts silences SPARC, a key bone matrix protein, reducing mineralization capacity [49]. Murine models with osteoblast-specific DNMT1 deletions further demonstrate DNMT1’s role in maintaining bone mass, as its loss leads to osteoporosis-like phenotypes [50].

In contrast, TET enzymes (TET1, TET2, TET3) promote DNA demethylation by oxidizing 5-methylcytosine, enabling transcriptional activation of osteogenic pathways. TET2, for instance, is critical for osteoclastogenesis; its deficiency in mice results in increased bone mass due to impaired osteoclast differentiation [50]. Aging reduces TET activity, leading to hypermethylation of anti-osteoclastogenic genes like OPG (osteoprotegerin), thereby shifting the RANKL/OPG balance toward bone resorption [51]. Conversely, TET1 enhances osteoblast differentiation by demethylating the BMP2 promoter, a pathway disrupted in aged MSCs [52]. Age-related oxidative stress and inflammation further exacerbate DNMT/TET imbalance. ROS inhibit TET activity, amplifying hypermethylation of osteogenic genes [53], while pro-inflammatory cytokines such as TNF-α upregulate DNMTs, silencing anti-resorptive genes like SOST (sclerostin) [54]. These shifts create a pro-osteoclastic environment and diminish bone formation, accelerating skeletal decline. Therapeutic strategies targeting DNMTs and TETs show promise in restoring bone homeostasis. DNMT inhibitors like 5-azacytidine reactivate silenced osteogenic genes in aged MSCs, enhancing osteoblastogenesis [55], while TET activators such as vitamin C improve bone repair by restoring demethylation of osteogenic promoters in preclinical models [56]. However, achieving tissue-specific modulation without systemic side effects remains a challenge. Collectively, age-related dysregulation of DNMTs and TETs underscores their pivotal roles in bone aging, offering a compelling rationale for epigenetic therapies to mitigate osteoporosis and skeletal fragility.

### 3.2 Histone Modifications and Chromatin Remodeling

Histone acetylation and deacetylation are pivotal epigenetic mechanisms in osteosarcopenia, regulating bone-muscle crosstalk through chromatin remodeling [57]. Class I/II histone deacetylases (HDACs) and NAD+-dependent sirtuins (SIRT1-7) control tissue-specific transcriptional programs, with their dysregulation accelerating age-related bone and muscle loss [58]. In muscle, HDAC4 suppresses MyoD expression, impairing myogenesis and regeneration, while HDAC1-mediated deacetylation at the Pax7 locus reduces satellite cell activation [59]. In bone, analogous HDAC activity disrupts osteogenic differentiation, though mechanisms remain under investigation. Conversely, sirtuins counter atrophy, SIRT1 represses Atrogin-1/MuRF1 in muscle and maintains osteocyte viability by deacetylating FOXO transcription factors [60], while mitochondrial SIRT3 sustains energy homeostasis in both tissues [61]. SIRT1 hypermethylation in senescent osteocytes driven by aging-related epigenetic shifts compromises its protective roles in bone remodeling [62]. Aging disrupts the HDAC-sirtuin equilibrium via NAD+ depletion [63], a hallmark of osteosarcopenia. Declining NAD+ levels impair SIRT1/SIRT3 activity, exacerbating mitochondrial dysfunction in muscle fibers and osteocytes [64], while inflammation upregulates HDAC2/3, repressing IGF-1 and PGC-1α in muscle [65]. These changes promote proteasomal degradation and suppress anabolic pathways, driving dual tissue atrophy. Therapeutically, HDAC inhibitors like vorinostat rejuvenate muscle stem cells and osteoprogenitors by restoring MyoD and osteogenic gene expression [66]. Nicotinamide riboside, an NAD+ booster, reactivates SIRT1/SIRT3, improving mitochondrial function in muscle and bone [67]. Resveratrol, a SIRT1 activator, enhances stress resistance in both tissues via FOXO signaling [68]. Tissue-specific delivery remains a challenge, but these strategies highlight the potential of epigenetic therapies to concurrently mitigate bone-muscle decline in osteosarcopenia.

**Figure 2.**
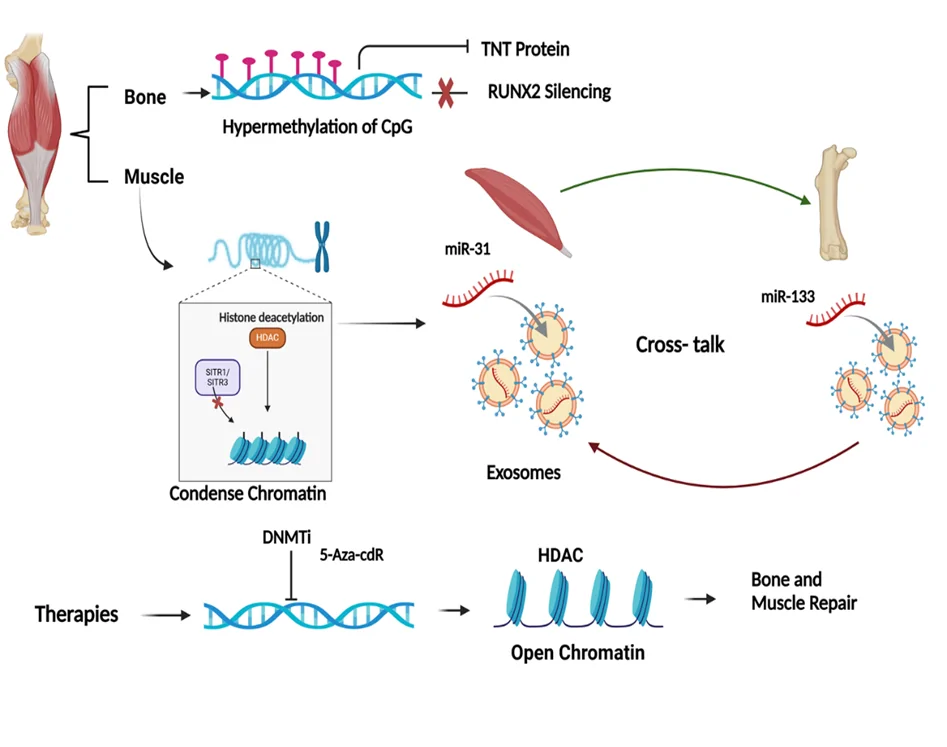
**Epigenetic Dysregulation in Bone and Muscle Aging**. This figure represents the epigenetic dysregulation DNA hypermethylation silences osteogenic genes (e.g., RUNX2), while HDAC overactivity impairs muscle repair. Exosomal miRNAs mediate bone-muscle crosstalk. Epigenetic therapies reverse these alterations.

### 3.3 Non-Coding RNAs in Bone-Muscle Crosstalk

Non-coding RNAs (ncRNAs), are critical mediators of bone-muscle crosstalk, with tissue-derived exosomes and epigenetic reprogramming playing pivotal roles in age-related osteosarcopenia [69]. Bone and muscle secrete ncRNAs including miRNAs, lncRNAs, and circRNAs via exosomes, enabling bidirectional communication that is disrupted during aging [70]. For example, muscle-derived miR-133, which suppresses osteogenic differentiation while promoting muscle atrophy, is elevated in aging skeletal muscle and inhibits bone formation by targeting RUNX2 and other osteogenic pathways [71]. Conversely, bone-derived exosomes enriched with miR-214 stimulate osteoclast activity, exacerbating bone resorption in osteoporotic conditions [72]. These ncRNAs are further dysregulated by SASP factors, which amplify inflammation and drive epigenetic alterations that impair tissue repair [73]. Notably, miR-31 and miR-206 exemplify the duality of ncRNA functions in musculoskeletal aging [74]. While miR-31 promotes osteoblast differentiation and is suppressed in osteoporosis, its exercise-induced upregulation in muscle may counteract bone loss Similarly, miR-206, essential for muscle repair, indirectly regulates osteoclast activity via RANKL inhibition, though its decline with age contributes to parallel declines in bone and muscle quality [75]. Senescence-associated epigenetic silencing of pro-regenerative ncRNAs (e.g., miR-31) and overexpression of catabolic ones (e.g., miR-133) create a vicious cycle, accelerating osteosarcopenia progression [76].

Therapeutic strategies targeting these pathways are emerging. Restoring miR-31 or inhibiting miR-133 and miR-214 could mitigate bone-muscle decline, while engineered exosomes delivering osteogenic miRNAs (e.g., miR-29b) or antagomirs against osteoclast-activating miRNAs (e.g., miR-214) show promise in preclinical models [77]. Future research must prioritize ncRNA networks that integrate mechanical loading, SASP-associated inflammation, and epigenetic dysregulation (Table 2.) By elucidating these mechanisms, ncRNA-based therapies could revolutionize treatment for osteosarcopenia, addressing both skeletal and muscular frailty through shared molecular pathways.

**Table 2:** Epigenetic Mechanisms in Bone and Muscle Aging.

Epigenetic Mechanism	Bone-Specific Effects	Muscle-Specific Effects	Key Regulator	Therapeutic targets
DNA Methylation	Hypermethylation of RUNX2, SPAR Reduced osteogenesis	Hypermethylation OPG, promoting resorption	DNMT3B, TET2	5-azacytidine, vitamin C
Histone Modifications	HDAC4-mediated repression of MyoD SIRT1/3 decline	HDAC1/4 suppression of Pax7 SIRT1 inhibition of Atrogin-1	HDAC1, HDAC4, SIRT1, SIRT3	Vorinostat, nicotinamide riboside
Non-Coding RNAs	miR-31 (pro-osteogenic), miR-133 (anti-osteogenic), exosomal miR-214	miR-206 (muscle repair), miR-483-5p (impaired metabolism), lncRNA DANCR (osteogenic)	miR-31, miR-133, miR-206, lncRNA MEG3	miRNA mimics/antagomirs, exosome therapies

## 4. Convergent Pathways: Epigenetics and Senescence in Osteosarcopenia

### 4.1 Shared Mechanisms Linking Osteocyte Senescence and Epigenetic Dysregulation

The interplay between osteocyte senescence and epigenetic dysregulation in osteosarcopenia is underpinned by shared pathways [78], notably inflammaging (Inflammaging, the age-associated chronic low-grade inflammation driven by pro-inflammatory cytokines (e.g., IL-6, TNF-α), amplifies tissue degeneration in bone and muscle) and metabolic dysfunction [79]. Inflammaging, the age-associated chronic low-grade inflammation, is driven in part by the SASP of osteocytes, which releases pro-inflammatory cytokines such as IL-6 and TNF-α, exacerbating tissue degeneration in bone and muscle [80]. Epigenetic silencing mechanisms, including DNA hypermethylation and histone deacetylation, further amplify this inflammatory milieu by repressing anti-inflammatory genes like SIRT1 and FOXO3, which normally mitigate oxidative stress and SASP activation [81]. For instance, age-related hypermethylation of SIRT1 promoters in osteocytes diminishes their capacity to suppress NF-κB signaling, creating a vicious cycle of inflammation and senescence [82].

Parallel to this, metabolic dysfunction marked by insulin resistance, mitochondrial inefficiency, and lipid accumulation acts as a common trigger for both senescence and epigenetic alterations [83]. Metabolic stressors like ROS and nutrient-sensing pathway disruptions e.g., mTOR, AMPK not only induce osteocyte senescence but also impair epigenetic modifiers such as DNMTs and TET enzymes, which rely on metabolites like α-ketoglutarate and NAD+ [84]. For example, mitochondrial dysfunction in aging skeletal cells reduces ATP and NAD+ availability, compromising HDAC activity and leading to aberrant gene expression linked to bone loss and muscle atrophy [85] (Figure 2). These convergent pathways highlight how metabolic disturbances epigenetically entrench senescent phenotypes, accelerating osteosarcopenia progression.

**Figure 3.**
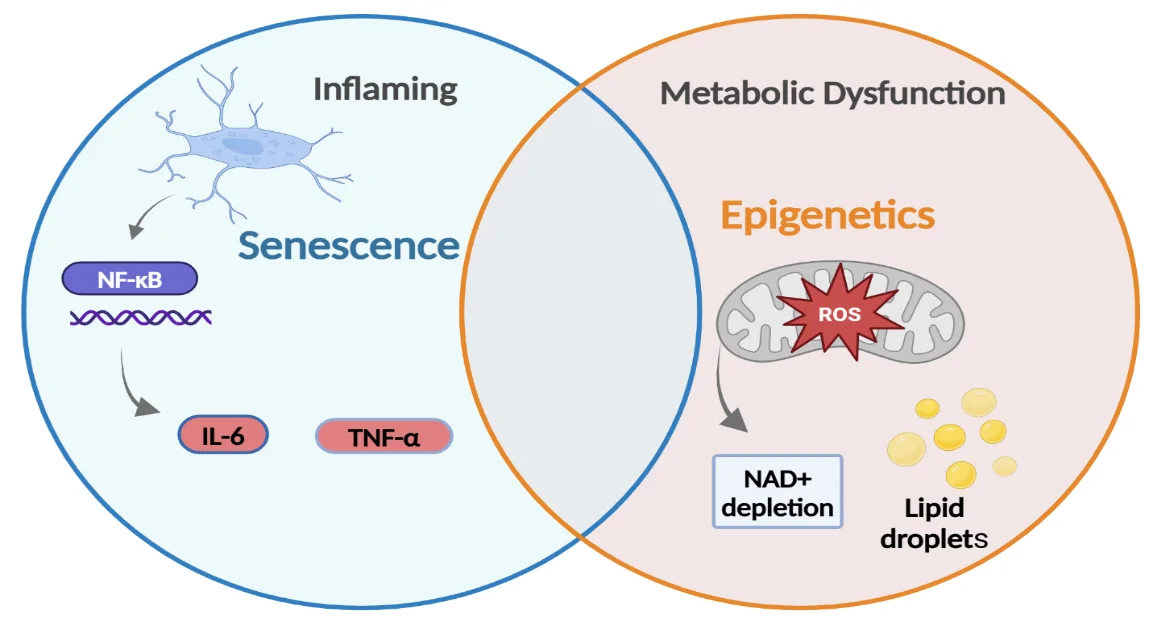
**Convergent Pathways of Senescence and Epigenetics**. This figure represents the convergent Pathways in Osteosarcopenia, senescence and epigenetics intersect via inflammation and metabolic dysfunction, perpetuating oxidative stress and chromatin instability. Biomarkers like methylation clocks reflect disease progression.

### 4.2 Osteosarcopenia a dual Pathology of Bone and Muscle

Osteosarcopenia, the concurrent deterioration of bone (osteopenia/osteoporosis) and muscle (sarcopenia), represents a critical geriatric syndrome that amplifies fracture risk, disability, and mortality in aging populations [1]. This dual pathology arises from shared biological mechanisms, including chronic inflammation, hormonal dysregulation, and cellular senescence, which disrupt the crosstalk between musculoskeletal tissues [86]. Bone and muscle, once viewed as mechanically linked but functionally distinct, are now recognized as endocrine organs that communicate via myokines e.g., myostatin, irisin and osteokines e.g., osteocalcin, sclerostin [1]. These molecules mediate muscle-bone interactions, coordinating adaptation to mechanical loading, energy metabolism, and tissue repair [87]. Dysregulation of this cross-talk, driven by age-related cellular dysfunction, underpins the parallel decline in bone density and muscle mass characteristic of osteosarcopenia [88].

### 4.3. Clinical Challenges in the Diagnostics and Staging of Osteosarcopenia

The diagnosis and staging of osteosarcopenia present significant clinical challenges due to the lack of standardized frameworks and the multifaceted nature of its pathophysiology. Emerging studies have explored biomarkers such as circulating irisin, TGF-β, and specific microRNAs (e.g., miR-133a) that may reflect concurrent bone and muscle deterioration through shared pathways like inflammation and oxidative stress [89]. However, their clinical translation is hampered by inconsistent assay methodologies, lack of population-specific reference ranges, and insufficient evidence linking biomarker levels to functional outcomes or disease progression. Current diagnostic tools, such as dual-energy X-ray absorptiometry (DXA) for measuring bone mineral density (BMD) and CT/MRI for assessing muscle mass, alongside functional metrics like grip strength and gait speed (per the EWGSOP2 criteria), are designed to evaluate bone and muscle health independently [90]. However, integrating these assessments into a unified diagnosis remains problematic. For instance, while osteoporosis is defined by a T-score ≤ -2.5, this metric does not account for muscle quality or function, whereas sarcopenia criteria often overlook bone health entirely [91]. This discordance leads to underdiagnosis, particularly in cases where bone loss (e.g., osteopenia with a T-score of -1.5) and muscle decline (e.g., reduced strength and slow gait) progress asymmetrically, failing to align with existing severity thresholds [92]. Additionally, there is no consensus on thresholds for classifying mild, moderate, or severe stages, creating ambiguity in prioritizing metrics like BMD, muscle strength, or composite scores [91]. Comorbidities such as diabetes or chronic kidney disease exacerbate these challenges by independently affecting both tissues, obscuring osteosarcopenia-specific progression. The absence of reliable biomarkers for dual-tissue decline, such as osteocalcin or myostatin, further limits accurate staging [93].

Efforts to address these gaps include developing composite indices that integrate BMD, muscle mass, strength, and functional assessments, with pilot studies proposing tiered staging systems [94]. Innovations in artificial intelligence (AI) aim to enhance risk stratification by combining imaging data with biomarkers, though universal adoption requires validation across diverse populations. Until standardized criteria emerge, clinicians must rely on multidisciplinary evaluations, balancing existing diagnostic tools with clinical judgment to guide personalized management. Future research must prioritize harmonizing diagnostic thresholds and refining staging frameworks to improve outcomes in this complex, interrelated condition.

## 5. Therapeutic Implications

### 5.1 Targeting Senescent Osteocytes

Accumulation of senescent cell like (osteocytes) in bone is a key driver of osteosarcopenia, exacerbating bone-muscle crosstalk dysfunction, chronic inflammation, and tissue degeneration [1]. While senolytics (e.g., dasatinib + quercetin) and senomorphics (e.g., rapamycin) demonstrate preclinical promise, their clinical translation faces osteosarcopenia-specific challenges. Senolytics, which target pro-survival pathways like BCL-2 and PI3K/AKT, risk off-tissue toxicity [95], dasatinib, a tyrosine kinase inhibitor, may impair immune function or worsen cardiovascular health, while quercetin’s broad antioxidative effects could inadvertently alter non-senescent cell survival [27]. Early-phase trials also report transient cytopenias and immune dysregulation, necessitating intermittent dosing to balance efficacy with hematopoietic safety [96]. A critical limitation is the lack of bone/muscle specificity, as systemic senescent cell clearance risks depleting beneficial populations, such as mechanosensitive pre-osteocytes or wound-healing fibroblasts. Senomorphics, though less cytotoxic, require chronic administration, increasing risks of immunosuppression (e.g., JAK/STAT inhibitors) or metabolic side effects (e.g., mTOR inhibitor-induced hyperlipidemia) [97] (Table 3). Their transient efficacy, seen with agents like metformin or ruxolitinib, demands sustained use to suppress SASP-driven inflammation and osteoclast activation [98]. Comorbidities further complicate translation, as preclinical models often neglect conditions like diabetes, where AMPK resistance in diabetic osteopathy may blunt metformin’s benefits [99]. Despite these hurdles, combining senolytics (e.g., dasatinib-quercetin) with senomorphics (e.g., rapamycin) synergistically improves bone density in aged mice by eliminating senescent cells and attenuating SASP-mediated osteoclastogenesis [100]. Emerging strategies, such as dual-action agents targeting osteocyte-specific markers (e.g., MMPs) or bone-muscle SASP components (e.g., IL-6), aim to enhance precision. Intermittent dosing and localized delivery systems (e.g., bone-targeted nanoparticles) are under exploration to mitigate off-target risks, though rigorous evaluation in comorbid models remains essential to address the multifaceted pathology of osteosarcopenia.

**Table 3:** Therapeutic Strategies Targeting Senescence and Epigenetics.

Therapy Type	Examples	Mechanism	Bone Benefits	Muscle Benefits	Status
Senolytics	Dasatinib + Quercetin	BCL-2/PI3K inhibition, ROS scavenging	Reduced osteoclast activity, improved BMD	Enhanced muscle regeneration	Phase II trials [101]
Senomorphics	Rapamycin, Ruxolitinib	Suppression of SASP (NF-κB, JAK/STAT)	Decreased IL-6/MMP-driven resorption	Reduced inflammation, improved repair	Preclinical [97]
Epigenetic Modulators	5-azacytidine (DNMTi), Vorinostat (HDACi)	Reactivate osteogenic genes (RUNX2), enhance histone acetylation (MYOD1)	Increased bone formation	Satellite cell activation, reduced atrophy	Preclinical/Phase I [102]
Lifestyle Interventions	Resistance exercise, Vitamin D	Mechanical loading (Wnt activation), epigenetic remodeling (MYOD1 demethylation)	Improved bone density	Muscle hypertrophy, strength gain	Clinical [103]

### 5.2 Epigenetic Intervention

Epigenetic therapies, though innovative, face significant translational barriers in addressing osteosarcopenia. DNMT inhibitors, such as 5-azacytidine, and HDAC inhibitors, like vorinostat, exhibit systemic toxicity that limits their clinical utility. DNMT inhibitors induce global hypomethylation, reactivating silenced tumor suppressor genes but risking oncogene activation (e.g., MYC) and causing myelosuppression or gastrointestinal toxicity, which are particularly problematic in non-cancer contexts like musculoskeletal aging [104]. Similarly, HDAC inhibitors disrupt acetylation-dependent processes across tissues, leading to pleiotropic effects such as arrhythmias, hepatotoxicity, and fatigue, complicating their use for age-related bone-muscle decline [12]. While combination therapies e.g., DNMT + HDAC inhibitors show synergistic preclinical promise in reversing age-related epigenetic silencing [105], their clinical application remains constrained by overlapping toxicities and a lack of tissue specificity. miRNA-based therapies, another promising avenue, confront delivery and safety challenges. miRNA mimics e.g., miR-34 and antagomirs e.g., anti-miR-133 suffer from poor stability and off-tissue uptake, as highlighted by the MRX34 trial [106], which was halted due to immune-related fatalities despite using liposomal delivery systems. Dose-dependent paradoxes further complicate outcomes, while miR-133 inhibition may enhance bone formation [107], excessive dosing risks exacerbating muscle fibrosis, undermining therapeutic balance in osteosarcopenia. Advances in nanoparticle delivery and chemical modifications (e.g., locked nucleic acids) aim to improve specificity, yet achieving bone-muscle selectivity remains elusive. For instance, antagomirs targeting osteoclast-promoting miRNAs (e.g., miR-214) could inadvertently impair muscle repair if broadly distributed. Current strategies prioritize localized delivery platforms and combinatorial approaches to mitigate risks, but rigorous safety profiling in age-related comorbidities is essential to advance these therapies for clinical use.

**Figure 4.**
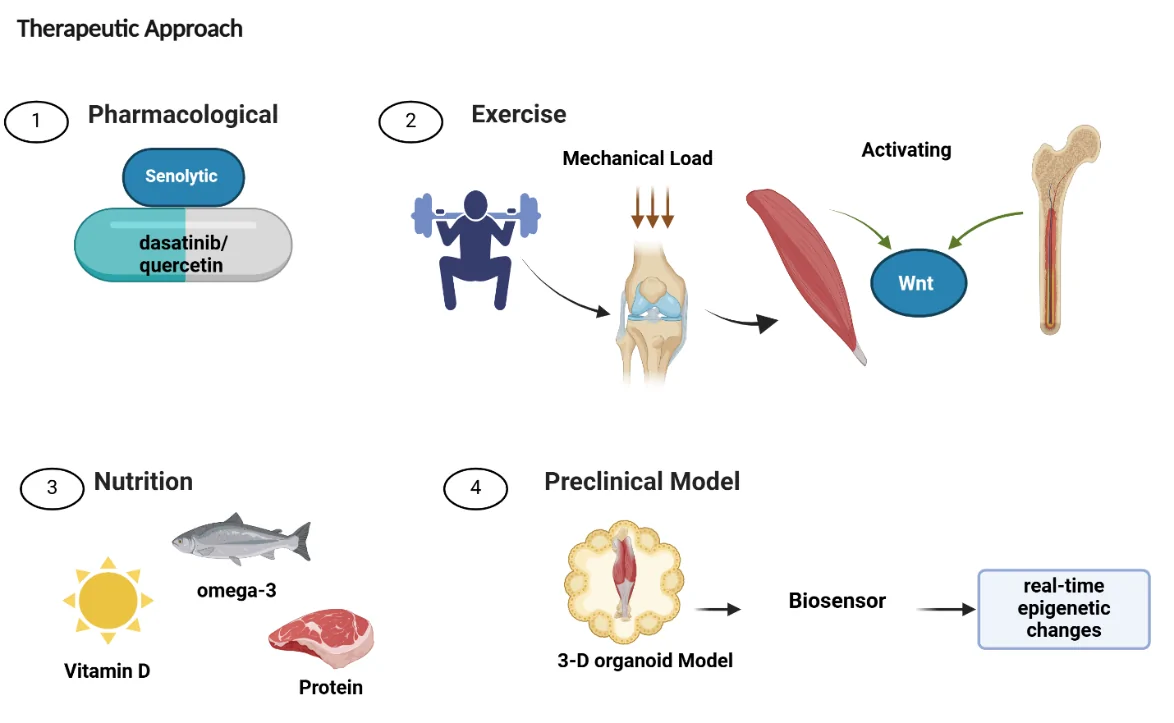
**Integrated Therapeutic Approaches**. This figure represents the integrated therapies like senolytics, epigenetic modulators, exercise, and nutrition synergize to restore bone-muscle crosstalk. Organoid models enable personalized intervention testing.

### 5.3. Integrated Approaches

The interdependence of bone and muscle, termed the “bone-muscle axis,” highlights the need for integrated strategies to address age-related osteosarcopenia. While dual-targeting pharmacotherapies and lifestyle interventions aim to synergistically improve musculoskeletal health, their efficacy is tempered by translational challenges. Bisphosphonates, widely used to preserve bone density by inhibiting osteoclast activity, exemplify the complexity of dual-tissue targeting [108]. Though they reduce inflammatory cytokines linked to bone resorption, their over-suppression of these signals may inadvertently impair muscle repair mechanisms, exacerbating atrophy in some patients. This paradox underscores the delicate balance required in modulating shared pathways like TGF-β, which regulates both bone remodeling and muscle fibrosis [109].

Alternative dual-targeting agents, such as selective androgen receptor modulators (SARMs), show promise in enhancing lean muscle mass and bone mineral density without androgenic side effects, though long-term safety data are pending [110]. Similarly, myostatin inhibitors improve mechanical loading on bone by promoting muscle growth, yet their systemic effects on non-muscle tissues remain poorly understood. Exercise and nutrition, though low-risk and foundational to musculoskeletal health, face limitations in real-world efficacy. Resistance training and weight-bearing exercises stimulate bone formation and muscle hypertrophy via mechanotransduction, while protein supplementation and vitamin D optimize tissue repair [111] (Figure 3). Despite progress, challenges persist, including the pleiotropic roles of pathways like Wnt/β-catenin critical for bone formation and muscle regeneration and the need for multidomain trials to validate synergies between pharmacotherapy, exercise, and nutrition in aging populations with comorbidities.

## 6. Future Directions

The interplay between cellular senescence and epigenetic regulation remains a critical yet underexplored frontier in understanding osteosarcopenia pathogenesis. To address critical gaps in understanding the interplay between senescence, epigenetics, and osteosarcopenia, researchers should prioritize humanized, comorbidity-integrated models and advanced methodologies that capture the complexity of human aging. Single-cell multi-omics approaches, such as integrating single-cell epigenomics scATAC-seq, scChIP-seq with transcriptomics, can map tissue-specific epigenetic changes e.g., DNA methylation shifts, H3K27me3 loss in senescent osteocytes and myocytes, resolving heterogeneity in chromatin remodeling and identifying drivers of osteosarcopenia. CRISPR-edited iPSC-derived bone-muscle organoids, engineered with patient-specific mutations e.g., p16INK4a, SIRT1, could model how epigenetic perturbations (e.g., HDAC inhibition) regulate senescence in diabetic or inflammatory microenvironments [112].

However, reproducibility challenges persist due to variability in iPSC differentiation protocols, donor-specific genetic backgrounds, and organoid maturation timelines, necessitating standardized protocols for cell sourcing, culture conditions, and phenotypic validation. To dissect causality, dCas9-based epigenetic editing tools (CRISPRa/i) could selectively modulate senescence-associated regulators (e.g., DNMTs, EZH2) in human cells, though off-target effects and inconsistent editing efficiency across cell types remain barriers to clinical translation.

**Table 4:** Future Research Directions and Methodologies.

Research Focus	Methodologies	Applications	Challenges
Single-Cell Multi-Omics	scATAC-seq, scChIP-seq, spatial transcriptomics	Map tissue-specific epigenetic changes in senescent osteocytes/myocytes	Data integration, computational complexity
CRISPR-Engineered Models	iPSC-derived organoids, p16-rtTA mice, base-edited diabetic models	Study senescence-epigenetics crosstalk in comorbidities (e.g., diabetes)	Model standardization, off-target effects
Senescence Biomarkers	Circulating miRNAs, SASP proteomics, senescence-associated methylation clocks	Early diagnosis, personalized therapy monitoring	Biomarker specificity, validation in diverse cohorts
Comorbidity-Inclusive Models	3D bioprinted co-cultures (senescent cells + adipocytes + immune cells)	Mimic osteosarcopenia in sarcopenic obesity	Reproducibility, scalability
Drug Synergy Testing	Senolytic + BET inhibitor combinations in organ-on-chip platforms	Identify synergistic therapies for bone-muscle crosstalk	Tissue-specific delivery, toxicity profiling

For biomarker discovery, ongoing longitudinal initiatives (e.g., OSTA (Osteosarcopenia Translational Alliance) and SOF (Study of Osteoporotic Fractures cohorts) are leveraging multi-omic profiling of liquid biopsies (cell-free DNA methylation, circulating miRNAs, SASP proteomics) to identify osteosarcopenia-specific signatures. Integrating epigenetic clocks and proteomics with machine learning distinguishes disease-specific biomarkers from aging processes. Humanized models (e.g., p16 reporter mice with diabetic senescent cells) and PET-MRI imaging validate biomarkers *in vivo*. CRISPR-edited organoids and diet-induced obesity models screen senolytic targets, though inconsistent methodologies hinder cross-study comparisons. Senolytic-epigenetic drug combinations (e.g., dasatinib + BET inhibitors) tested in organ-on-chip platforms face tissue-specific delivery challenges. Population-scale epigenome-wide studies (EWAS) paired with Mendelian randomization and digital twins enable personalized therapies, while consortia (e.g., HIPPO) standardize models and address comorbidity complexity. Polypharmacy approaches in comorbid mice assess drug interactions. Prioritizing human pathophysiology and standardizing biomarkers across diverse cohorts are critical for advancing osteosarcopenia therapies. Future efforts must ensure reproducibility in multi-omics integration and clinical translation, as outlined (Table 4).

## 7. Conclusion

Osteosarcopenia, the intertwined decline of bone and muscle with aging, epitomizes the convergence of gerontology, musculoskeletal biology, and epigenetics. While cellular senescence and epigenetic dysregulation marked by SASP-driven inflammation, DNA methylation drift, and chromatin instability are established as central drivers, the absence of formal clinical guidelines impedes translational progress. This gap stems from three key challenges. First, variability in diagnostic criteria, where osteosarcopenia is variably defined as the coexistence of osteoporosis (T-score ≤-2.5) and sarcopenia (EWGSOP2 criteria) or stricter composite thresholds. Second, discrepancies in population-specific cutoffs, as ethnicity, sex, and comorbidities (e.g., diabetes, chronic kidney disease) alter bone-muscle decline trajectories. Third, limited longitudinal data to map intersecting rates of bone loss and muscle atrophy, hindering evidence-based staging. Mechanistically, shared pathways like Wnt/β-catenin suppression, mitochondrial dysfunction, and nutrient-sensing dysregulation offer therapeutic targets, yet the crosstalk between senescence and epigenetics particularly how tissue-specific epigenetic landscapes dictate osteosarcopenia susceptibility remains poorly resolved. Clinically, integrating multimodal biomarkers (e.g., senescence-associated DNA methylation signatures, exosomal ncRNAs, and advanced imaging) could revolutionize risk stratification. However, biomarker validation is stymied by diagnostic heterogeneity and the lack of standardized thresholds. Similarly, senolytics and epigenetic modulators (e.g., HDAC inhibitors, miR-133 antagomirs), though promising, require optimization to balance tissue specificity and off-target risks. Preclinical models must evolve to reflect human aging complexity, incorporating comorbidities, cellular crosstalk, and dynamic senescence-epigenetics interactions. Longitudinal studies tracking bone-muscle trajectories in diverse populations are critical to delineate staging frameworks and refine therapeutic windows. Interdisciplinary collaboration is paramount to bridge molecular insights with clinical implementation, ensuring therapies address phenotypic heterogeneity. By targeting upstream drivers like NAD+ depletion or SASP amplification, future strategies could shift from symptomatic care to precision prevention, preserving mobility in aging populations. Realizing this vision demands harmonized diagnostic criteria, validated biomarkers, and geroscience-driven trials, ushering in an era of integrated musculoskeletal care.

## Data sharing

Data will be available on request

## Transparency

The corresponding author HY and first author SN affirm that the manuscript is an honest, accurate, and transparent account of the reported study. No aspects of the study have been omitted, and discrepancies from the study as initially planned have been explained.
